# Wild-type huntingtin regulates human macrophage function

**DOI:** 10.1038/s41598-020-74042-8

**Published:** 2020-10-14

**Authors:** Grace C. O’Regan, Sahar H. Farag, Gary R. Ostroff, Sarah J. Tabrizi, Ralph Andre

**Affiliations:** 1 UCL Huntington’s Disease Centre, Department of Neurodegenerative Disease, UCL Queen Square Institute of Neurology, University College London, London, WC1N 3BG UK; 2 Program in Molecular Medicine, University of Massachusetts Medical School, 373 Plantation Street, Two Biotech, Suite 113, Worcester, MA 01605 USA; 3 UK Dementia Research Institute at UCL, UCL Queen Square Institute of Neurology, University College London, London, WC1N 3BG UK

**Keywords:** Neuroimmunology, Monocytes and macrophages

## Abstract

The huntingtin (HTT) protein in its mutant form is the cause of the inherited neurodegenerative disorder, Huntington’s disease. Beyond its effects in the central nervous system, disease-associated mutant HTT causes aberrant phenotypes in myeloid-lineage innate immune system cells, namely monocytes and macrophages. Whether the wild-type form of the protein, however, has a role in normal human macrophage function has not been determined. Here, the effects of lowering the expression of wild-type (wt)HTT on the function of primary monocyte-derived macrophages from healthy, non-disease human subjects were examined. This demonstrated a previously undescribed role for wtHTT in maintaining normal macrophage health and function. Lowered wtHTT expression was associated, for instance, with a diminished release of induced cytokines, elevated phagocytosis and increased vulnerability to cellular stress. These may well occur by mechanisms different to that associated with the mutant form of the protein, given an absence of any effect on the intracellular signalling pathway predominantly associated with macrophage dysfunction in Huntington’s disease.

## Introduction

Huntingtin (HTT) is best known for its role in the autosomal dominant inherited neurodegenerative condition, Huntington’s disease, where a polyglutamine-encoding CAG repeat in exon 1 of the gene is expanded over a fully-penetrant disease-causing threshold^1^. The protein is expressed ubiquitously but is associated mostly with its effects on the central nervous system (CNS) during Huntington’s disease pathogenesis. Compared to extensive research into the toxic gain-of-function properties of the mutant protein (mHTT), studies of the function of the wild-type form of the protein (wtHTT) have been less prevalent and, therefore, the full scope of its normal function(s) remain incompletely defined. Nevertheless, much is known about wtHTT’s expression, structure, post-translational modification and binding partners^2^, and some broad biological functions have been described, including essential roles in gastrulation^3–5^ and the embryonic development of the CNS^6–11^. Beyond development, wtHTT is known to have anti-apoptotic effects by means of regulating caspase activity, whereby its overexpression is beneficial in terms of reducing the loss of selectively vulnerable cells in models of Huntington's disease^12–15^;  conversely; its absence is associated with worsened disease phenotypes, survival rates and cell vulnerability^16–18^. Neuroprotective effects of wtHTT are also observed in, for example, mouse models of ischaemia and traumatic brain and spinal cord injuries^19^. Interactions of wtHTT with various components of the cytoskeleton underlie key roles in vesicular trafficking and cell adhesion^20–23^, and regulation of transcription by various means has also been described^24–26^. Wild-type HTT’s roles in both vesicle trafficking and transcription have received particular attention in the context of the production and transport of the neurotrophic factor, BDNF^27,28^.

In the immune system, various abnormalities have been shown in Huntington’s disease gene carriers, both in the CNS and the periphery^29–37^. There is evidence, moreover, that aspects of the innate immune system may play a modifying role in disease pathogenesis^38–43^. Myeloid cells comprising circulating monocytes and tissue-resident macrophages, including microglia in the CNS, are the likely effector cells of these innate immune system phenotypes. Such cells are hyper-reactive in response to Toll-like receptor (TLR) agonists such as lipopolysaccharide (LPS)^31,44^ and exhibit functional deficits in their migratory and phagocytic capabilities^44,45^ by means of the cell-autonomous effects of mHTT expression on intracellular signalling and/or gene expression^46–48^.

By comparison, whether wtHTT has any role in the normal function of monocytes and macrophages remains unexplored. The use of β-1,3-D-glucan-encapsulated anti-*HTT* siRNA particles (GeRPs) in prior studies involving monocyte-derived macrophages from Huntington’s disease patients showed apparent effects on cytokine release following stimulation whether it be the mutant or the wild-type *HTT* allele that was specifically targeted^49^, with some indication of a similar effect in the control cells from healthy individuals^46,49^. Given the roles wtHTT might play in cytoskeleton remodelling, intracellular trafficking and transcription, it is plausible that a yet undescribed role for wtHTT could exist in cells of the innate immune system. The present work aimed, therefore, to investigate this further, by examining the effects of lowering the expression of wtHTT on the function of primary monocyte-derived macrophages from healthy, non-disease human subjects. This is relevant in defining what might be a novel role for this protein in cells beyond the CNS and in the normal function of the innate immune system.

## Results

### Cytokine release by primary human macrophages is regulated by wtHTT expression levels

**Figure 1:**
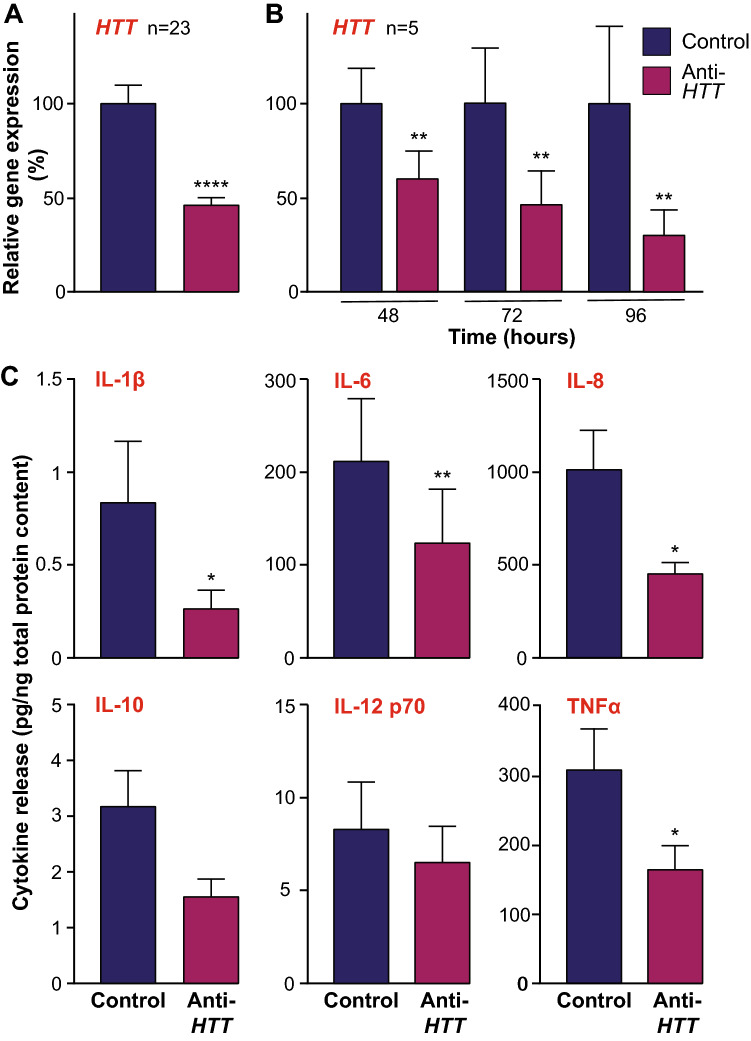
Wild-type HTT regulates cytokine release by human macrophages. Primary macrophages were derived from blood monocytes harvested from a cohort of healthy human subjects and cultured for treatment with either anti-*HTT* or scrambled siRNA-containing GeRPs. (**A**) To confirm knock-down of *HTT* expression, cells were harvested and subject to qPCR analysis. This showed a significant lowering of *HTT* expression 48 h post-treatment in the anti-*HTT* treated cells compared to those treated with the scrambled siRNA (n = 23; ****p < 0.0001). (**B**) To demonstrate the maintenance of lowered HTT expression over periods of time useful for subsequent experiments, the experiment was repeated with measures made at 48, 72 and 96 h post-treatment. This demonstrated that HTT lowering was fully maintained in culture for at least four days (n = 5; **p < 0.01). (**C**) Supernatants were harvested from cultures of GeRP-treated primary macrophages following stimulation with 2 µg/ml LPS and 10 ng/ml IFNγ for 24 h, and analysed by multi-plex ELISA for levels of cytokines relative to total culture protein content, including IL-1β, IL-6, IL-8, IL-10, IL-12 p70 and TNFα. This showed significantly reduced levels of IL-1β, IL-6, IL-8 and TNFα in the anti-*HTT* treated cultures (n = 7–10; *p < 0.05, **p < 0.01). Data are presented as mean ± SEM, analysed by paired two-tailed Student’s *t*-test.

HTT-lowering in primary human macrophages was achieved by treatment with anti-*HTT* siRNA-containing GeRPs, as described previously^46^. To confirm *HTT* lowering, parallel wells of cultured cells from each subject were treated with particles carrying anti-*HTT* (knockdown) or scrambled (control) siRNA sequences. RT-qPCR showed that macrophages treated with anti-*HTT* siRNA consistently expressed *HTT* at levels that were reduced by 50–60% (****p < 0.0001; Fig. 1A), in line with previous observations^46^; prior work has shown this correlates with a ~ 50% reduction in HTT protein expression levels^49^. To ensure *HTT* expression levels remained knocked-down throughout the likely time-course of further experiments, the lowering effects of anti-*HTT* GeRPs over time were investigated in a sub-set of samples. RT-qPCR showed again that macrophages treated with anti-*HTT* siRNA had consistently reduced *HTT* expression levels and that these levels remained significantly lowered for the full 96 h duration of the experiment (**p < 0.01; Fig. 1B). Activation of the macrophages was achieved by treatment with LPS and IFNγ, also as described previously^46^. To demonstrate this here, supernatants were harvested from macrophages either stimulated with LPS and IFNγ or vehicle alone for 24 h and assessed for the secreted levels of six cytokines, IL-1β, IL-6, IL-8, IL-10, IL-12 p70 and TNFα, by multiplex ELISA. This showed that each of the cytokines were induced by stimulation with LPS and IFNγ, as expected (Supplementary Fig. S1).

Next, in a cohort of new samples, the effects of HTT-lowering on cytokine expression by stimulated primary human macrophages was assessed. Following treatment with anti-*HTT* or scrambled siRNA-containing GeRPs, macrophages were stimulated with LPS and IFNγ for 24 h. Supernatants were harvested and assessed for the secreted levels of six cytokines, as before, in this case demonstrating that four of the cytokines measured, IL-1β, IL-6, IL-8 and TNFα, each of which are considered pro-inflammatory, were secreted to a significantly reduced degree following *HTT*-lowering (**p < 0.01, *p < 0.05; Fig. 1C). The anti-inflammatory cytokine, IL-10, appeared also to be secreted at lower levels, to a degree that was near-significant (*p* = 0.0503). This demonstrates that release of these cytokines by macrophages is in some way regulated by wtHTT, a novel function of this protein in these cells.

### The effects of wtHTT on cytokine release by primary human macrophages are observed at the level of gene transcription

The reduced levels of pro-inflammatory cytokines released by primary human macrophages in which *HTT* expression is lowered could occur by one or more of several mechanisms. Given previous data showing that expression of the LPS receptor, TLR4, is not affected by HTT-lowering in these cells, it is plausible, for example, that the signalling pathways leading to expression of the genes encoding pro-inflammatory cytokines are altered, or that wtHTT acts at the level of their intracellular trafficking and release. Should cytokine gene expression be diminished upon *HTT* lowering, this would suggest that the regulation of cytokine secretion by wtHTT has at least some basis in transcription. Alternatively, if levels of cytokines are elevated inside the cells, their accumulation there might infer that normal intracellular flux of these proteins is impeded because of a diminished role for wtHTT in their trafficking and release. If this was observed in the absence of any difference in cytokine gene expression, it could be reasoned that the reduced levels of cytokine secretion resided *solely* in interactions of wtHTT with the intracellular trafficking and release machinery. Conversely, of course, it is possible that wtHTT could have parallel roles in macrophages in cytokine gene expression and trafficking and release alike.

**Figure 2:**
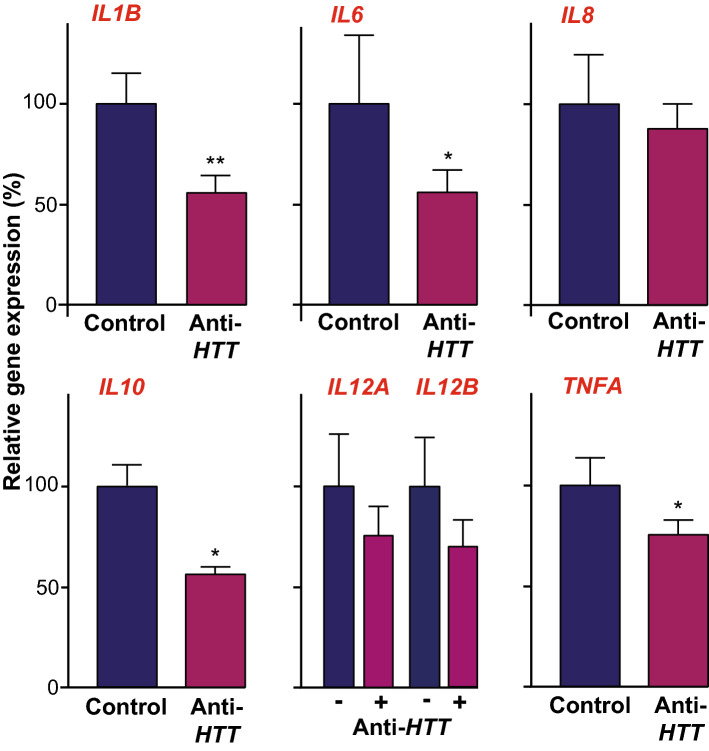
Wild-type HTT lowering reduces expression of genes encoding cytokines in human macrophages. Primary macrophages treated with either anti-*HTT* or scrambled siRNA-containing GeRPs and stimulated with 2 µg/ml LPS and 10 ng/ml IFNγ for four hours, were harvested for qPCR quantification of the expression of genes encoding cytokines whose release was measured previously (IL12 p70 exists as a heterodimer comprising monomers encoded by two separate genes, *IL12A* and *IL12B*). This showed significantly reduced levels of *IL1B*, *IL6, IL10* and *TNFA* expression levels in the anti-HTT treated cultures (n = 3–5; *p < 0.05, **p < 0.01). Data are presented as mean ± SEM, analysed by paired two-tailed Student’s *t*-test.

Therefore, primary human macrophages treated with anti-*HTT* or scrambled siRNA-GeRPs were stimulated with LPS and IFNγ for 4 h. Lysates of each sample were assessed by RT-qPCR for the expression of the genes, *IL1B*, *IL6, IL8, IL10, IL12A, IL12B* and *TNFA*, encoding the cytokines whose release was measured previously. This showed that the *IL1B*, *IL6, IL10* and *TNFA* gene transcripts were significantly reduced in the HTT-lowered condition (**p < 0.01, *p < 0.05; Fig. 2). These data suggest that wtHTT exerts its effects on cytokine release by macrophages, at least in part, by acting on the expression of the genes encoding them and/or signalling pathways upstream of this.

**Figure 3:**
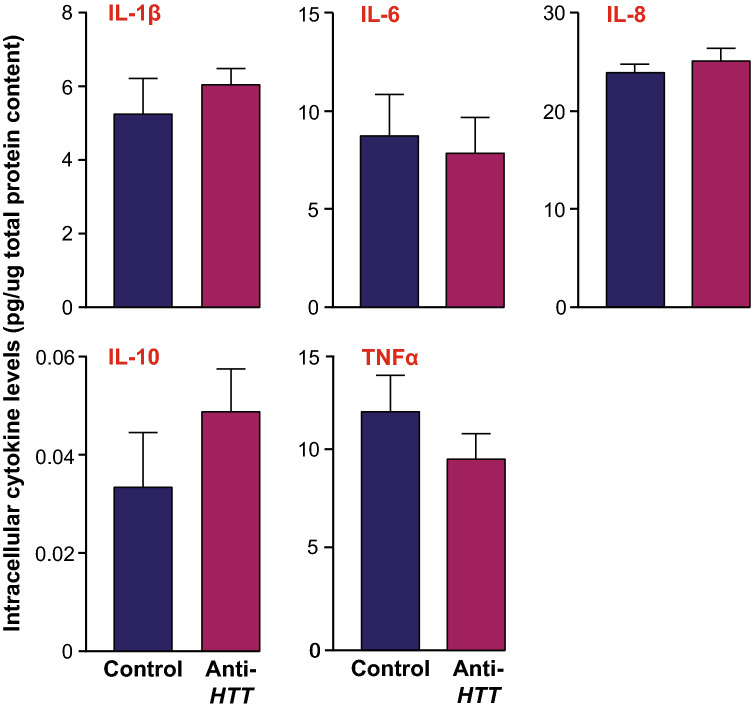
Intracellular levels of cytokines in human macrophages are not affected by wtHTT lowering. Primary macrophages treated with either anti-*HTT* or scrambled siRNA-containing GeRPs and stimulated with 2 µg/ml LPS and 10 ng/ml IFNγ for 24 h, were harvested for lysis and measurement of intracellular levels of cytokines whose release was shown previously to be affected by HTT lowering. Multi-plex ELISA analysis of steady-state intracellular IL-1β, IL-6, IL-8 and TNF-α levels relative to total culture protein content showed that they were not significantly affected by HTT lowering (n = 4). Data are presented as mean ± SEM, analysed by paired two-tailed Student’s *t-*test.

In parallel, primary human macrophages were treated with anti-*HTT* or scrambled siRNA-GeRPs and stimulated with LPS and IFN-γ for 24 h. In this instance, lysates of each sample were assessed by multiplex ELISA for intracellular levels of IL-1β, IL-6, IL-8, IL-10 and TNFα. This did not show any differences in levels caused by HTT-lowering (Fig. 3). That there was no evidence of an intracellular accumulation of the cytokines suggests that their normal flux through the cell prior to secretion was maintained. Taken together, these data suggest that wtHTT’s role in cytokine secretion does not reside solely in interactions with the intracellular trafficking and release machinery, rather that it exerts its effects on cytokine release by macrophages by acting, at least in part, on the expression of the genes encoding them and/or signalling pathways upstream of this.

### Wild-type HTT does not act on the NFκB pathway to regulate cytokine release by primary human macrophages

Previous work has shown that increases in cytokine release by mHTT-expressing primary human monocytes and macrophages are partially explained by an interaction of mHTT with the NFκB pathway, with elevated levels of NFκB translocation to the nucleus observed following LPS stimulation^46^. It follows, therefore, that wtHTT may act on this pathway similarly and that lowering its expression levels in macrophages might reduce NFκB activity in a manner concomitant to their release of pro-inflammatory cytokines.

**Figure 4:**
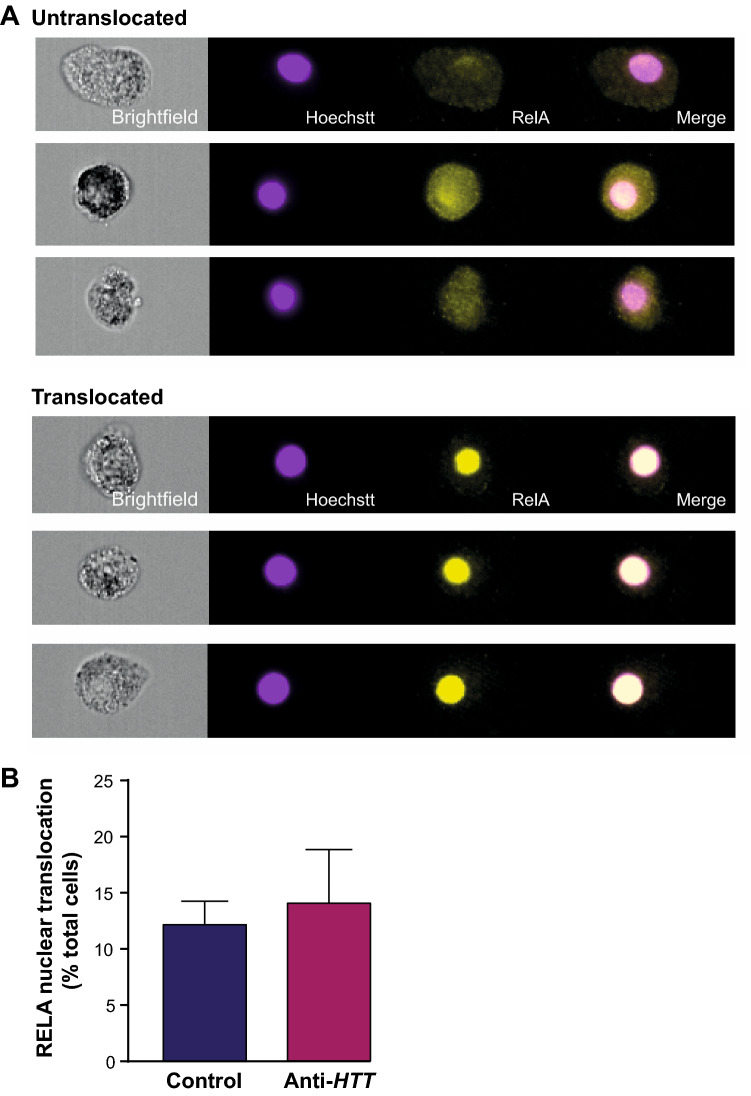
NFκB activity is not affected by wtHTT lowering in human macrophages. Primary macrophages treated with either anti-*HTT* or scrambled siRNA-containing GeRPs and stimulated with 2 µg/ml LPS and 10 ng/ml IFNγ for 45 min, were analysed by imaging flow cytometry for RELA translocation into the cell nucleus. RELA is one subunit of the heterodimeric complex that comprises the classical/canonical form of NFκB. (**A**) Example images are shown of cells in which RELA had or had not translocated into the nucleus as determined by overlapping RELA (yellow) and Hoechst (purple). (**B**) There was no significant difference in the percentage of total cells showing RELA translocation into the nucleus (n = 4). Data are presented as mean ± SEM, analysed by paired two-tailed Student’s *t*-test.

Primary human macrophages were treated with anti-*HTT* or scrambled siRNA-GeRPs and stimulated with LPS and IFN-γ for 45 min, a length of time in which NFκB translocation has been shown to peak in these cells^46^. NFkB is a heterodimeric complex, comprising in its classical/canonical form, RELA (p65) and p50 subunits. Levels of RELA translocation were assessed using imaging flow cytometry, which demonstrated no difference in NFκB activity in the HTT-lowered state; indeed, if anything it was slightly although not significantly higher (Fig. 4). This shows that the actions of wtHTT on cytokine release by macrophages do not have their basis in this pathway and that those of mHTT are an independent, gain-of-function mechanism of the disease-associated protein.

### Wild-type HTT expression levels affect phagocytosis and responses to stress by primary human macrophages

**Figure 5:**
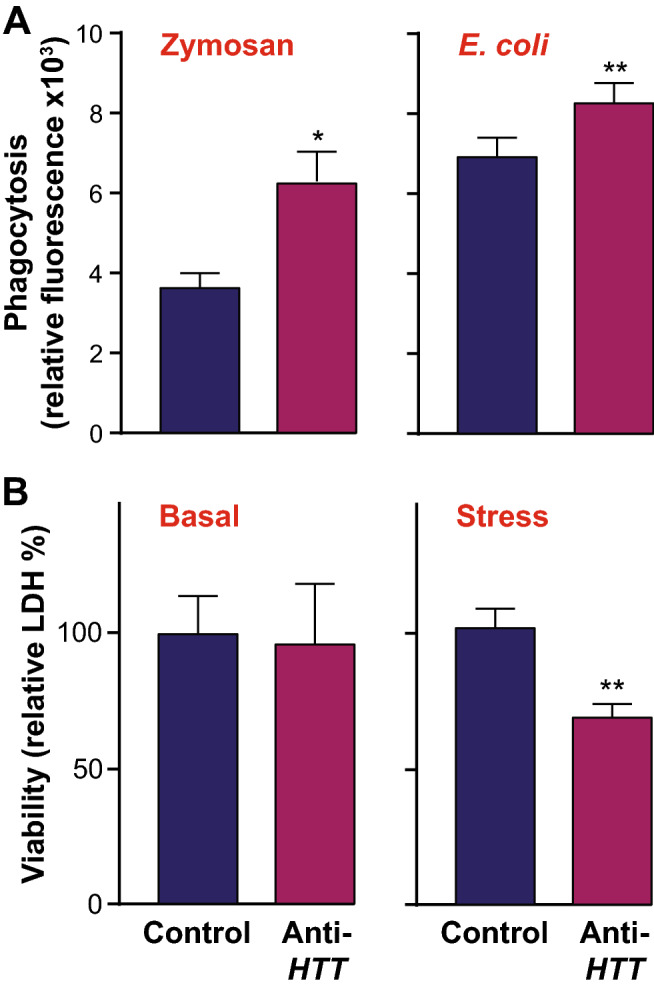
Wild-type HTT lowering increases phagocytosis by human macrophages and their susceptibility to stress. (**A**) Phagocytic activity, assessed using either zymosan or *E. coli* pHrodo beads, was significantly increased in primary human macrophages treated with anti-*HTT* siRNA-GeRPs compared to those treated with scrambled siRNA (n = 4–5; *p < 0.05, **p < 0.01). (**B**) Viability as assessed by LDH release of primary human macrophages treated with anti-*HTT* siRNA-GeRPs was not altered compared to those treated with scrambled siRNA (n = 6), unless the cells were exposed to a stress, bafilomycin A1, for nine hours (n = 3; **p < 0.01). Data are presented as mean ± SEM, analysed by paired two-tailed Student’s *t*-test.

Macrophages are well-known for their ability to phagocytose foreign material and cell debris as part of their role in immune responses. To determine whether wtHTT affects macrophage function beyond the release pro-inflammatory cytokines, phagocytosis was assessed in cells stimulated with either zymosan or *E. coli* bioparticles. This showed that phagocytosis was elevated in primary human macrophages under conditions of HTT-lowering by means of anti-*HTT* siRNA-GeRPs compared to control (**p < 0.01, *p < 0.05; Fig. 5A).

Finally, given the known protective effects of wtHTT, at least in the context of neural cells, the viability of primary human macrophages under HTT-lowered conditions was assessed. The cells were treated with anti-*HTT* or scrambled siRNA-GeRPs and viability assessed by LDH assay. This showed no significant difference (Fig. 5B). However, following introduction of a cell stressor, the autophagy inhibitor bafilomycin A1, a significant difference in the HTT-lowered condition emerged (*p < 0.05). Taken together, this suggests that wtHTT regulates various aspects of the function of primary human macrophages and their responses to stimulation or stress.

## Discussion

Previous data indicated the possibility of a role for wtHTT in cytokine release from monocytes and macrophages, whether they carry the Huntington’s disease mutation or not^46,49^. In the present study, the cytokine profiles ex vivo of a large number of macrophage samples from normal, healthy individuals treated with either anti-*HTT* or scrambled siRNA GeRP-treated were obtained. This showed a clear reduction in the LPS-mediated release of pro-inflammatory cytokines in the HTT-lowered state, confirming a novel role for wtHTT in the function and responses of human macrophages. There was no apparent difference in the extremely low, tonic levels of cytokines secreted by macrophages in the absence of any stimulation, either due to a lack of sensitivity in the assays used, or because the effects of wtHTT relate to the cells’ function only when they are activated.

There are several means by which wtHTT could exert regulatory effects on cytokine expression and release. For example, given documented roles of HTT in protein and vesicle trafficking and secretion^20,50,51^, it is plausible that HTT-lowering impairs this function in human macrophages to reduce pro-inflammatory cytokine release. It may also be the case that HTT-lowering reduces the expression of the cytokines themselves. There is ample evidence, for example, showing HTT has the capacity to affect transcription, either by direct interaction with transcription factors^28^, or with chromatin modifying proteins^52^. Alternatively, HTT may interact with upstream intracellular signalling pathways, the effect of which is to alter transcription^46,49^. By any of or all these means, wtHTT could alter the expression of genes encoding pro-inflammatory cytokines. Here, expression of genes encoding pro-inflammatory cytokines, at least those encoding IL-1β, IL-6, IL-10 and TNFα, was significantly reduced in activated macrophages in which wtHTT expression levels had been lowered. It seems, therefore, that wtHTT regulates the expression of genes encoding pro-inflammatory cytokines in stimulated macrophages in a manner reflected by the eventual release of the cytokines themselves from the cells.

Any such differences were not accompanied by altered intracellular levels of the cytokines. Given that an effect of wtHTT on the trafficking of cytokines to the cell surface under stimulated conditions might be expected, if all else is equal, to be revealed when wtHTT expression levels are lowered by way of a build-up of their intracellular levels, these data suggest the absence of an overt role for wtHTT in exocytic trafficking of these cytokines. However, whilst intracellular levels of the cytokines were not elevated when wtHTT expression was reduced, neither were they diminished. It might be reasoned that if expression of the gene encoding a secreted protein goes down and so does eventual release of the protein too, then levels of the protein in the intracellular compartments through which it moves, from its initial translation to final secretion, should also be reduced. Yet from what is known about how cytokines are released from macrophages, it is entirely plausible that this is not the case. Whilst many immune cells store cytokines for rapid release in compartments such as secretory granules, macrophages do not have these granules and instead after cell activation, cytokines must be synthesised and secreted as quickly as possible, either via the constitutive secretory pathway or by other, non-conventional means^53–55^. To accommodate the need to transport and release large volumes of newly synthesised cytokines after activation, therefore, expression of key components of the cellular machinery and carriers involved are upregulated too.

Indeed, there are plentiful examples of how expression of components of the cytokine secretion machinery is regulated in a manner that reflects the activation state of the cells. For example, the golgin, GOLGA4/p230, which regulates trafficking to and from the trans Golgi network, is selectively regulated to enhance cytokine transport^55,56^, as is RAB6, which regulates GOLGA4/p230-labelled carriers of TNFα^57^. A number of lipid metabolic genes are also regulated in this way, including those for CCTα, PLD1 and C/EPT, with lipids having an active role in the biogenesis of carriers and organising trans Golgi exit domains^58^. Similarly, as the cargo transitions from the Golgi to recycling endosomes, levels of SNAREs and other proteins that regulate this process and that of the subsequent fusion of such endosomes with the plasma membrane, are dependent on the activation state of the cell^53,59–61^. Surface delivery is also regulated by more RABs, including RABs 11 and 37; expression of these too is regulated by cell activation^59,62^. These data illustrate how expression of the cellular machinery needed to secrete cytokines can be regulated by the same signals that regulate expression of the cytokines themselves. This suggests that when the flux of a particular cytokine through the cell is altered (ie more or less is being made but it is being transported out of the cell at a higher or lower rate too), the actual intracellular level of that cytokine detected at a particular cross-sectional timepoint might not be, which is in line with our results.

Taken together, an interaction of wtHTT with intracellular signalling pathways and/or transcriptional machinery that are responsive to TLR-mediated activation of macrophages is a putative mechanism of action best supported by the present data. That’s not to say that wtHTT does not regulate other aspects of cytokine secretion, nor that there might be redundancy in the system or compensatory mechanisms to ensure that intracellular cytokine flux is maintained when wtHTT is lowered. Indeed, the finding that intracellular levels of cytokines are not reduced while extracellular levels are does not rule out effects on trafficking and release*.* It should also be noted, for example, that whilst IL-8 secretion is reduced when wtHTT expression is lowered, expression of the *IL8* gene is not, or least not to a statistically significant degree. This may then reflect an undefined role for wtHTT in its intracellular trafficking, or it may be an example of a protein whose altered expression is not reflected at the gene transcript levels. Furthermore, different cytokines are regulated and processed in ways that, despite much overlap, differ from one another. Nevertheless, if an interaction of wtHTT with the cytokine trafficking and secretion machinery does exist, then often it does so in the context of differences in the levels of transcription of the genes encoding them.

Expression of mHTT in human macrophages is associated with an interaction with a key component, IKK, of the intracellular signalling cascade leading to the activation of the transcription factor, NFκB, which is a potent driver of TLR-mediated pro-inflammatory gene expression^46^. It is plausible, therefore, that wtHTT acts on the same pathway to regulate the expression of pro-inflammatory cytokines, with the mutant protein presumably potentiating this effect. However, translocation of NFκB into the nucleus following stimulation of macrophages was not diminished by the lowering of wtHTT expression levels. This suggests, critically, that the mechanism by which wtHTT participates in the expression of pro-inflammatory cytokines is distinct to the pathological gain of function activation of the NFκB pathway that occurs in Huntington’s disease monocytes and macrophages.

Another key cellular function of macrophages is phagocytosis. In Huntington’s disease, levels of phagocytic activity are elevated in primary macrophages^45^. This is mirrored here by a similar phenotype when wtHTT expression levels are lowered, in a manner encompassing multiple activators of phagocytosis. This suggests that the disease phenotype may, at least in part, be caused by a loss of function of wtHTT. Such an effect may be orchestrated through known interactions of HTT with the cytoskeleton. For example, HTT has a role in nuclear actin reorganisation that is impaired upon HTT-lowering^63^. More generally, on the continuum that is macrophage polarisation states, historically described in terms of polarised ‘M1′ and ‘M2′ phenotypes, the effects of HTT-lowering on increasing phagocytosis under basal conditions and reducing pro-inflammatory cytokine release in response to LPS and IFNγ is suggestive of a role for wtHTT in determining macrophage fate on this spectrum^64,65^. In turn therefore, in the Huntington’s disease-state, the replacement of 50% of wtHTT expression by mHTT may instead skew this effect on macrophage polarisation towards a more inflammatory phenotype.

Wild-type HTT-dependent shifts in the functional state of macrophages were accompanied by differences in the cells’ vulnerability to stress. Specifically, reduced wtHTT expression in primary human macrophages was associated with reduced viability when under stress. In the absence of a stress, there was no discernible effect of wtHTT-lowering on the cells’ viability. This reduced ability to cope with cellular stressors under fits with known roles for wtHTT in coordinating cell stress responses, such as regulating caspase 3 activity and the recruitment of caspase 8^12,13,66,67^. Indeed, it may be that the impact of wtHTT-lowering on macrophage viability under stressed conditions has much in common with known interactions of wtHTT with stress response pathways in neural cells in Huntington’s disease, whereby a number of disease phenotypes are worsened in the absence of the presumably protective wtHTT protein^14,19,25,28^.

Taken together, therefore, these data establish a novel role of wtHTT as a regulator of macrophage health and function. Understanding the mechanisms by which this occurs may offer new means of modulating levels of inflammation in immune system disorders. Targeting HTT by means of lowering its expression is already being tested in the context of Huntington’s disease therapy^68,69^. Indeed, given this, and that evidence to date suggests much overlap in the effects of the HTT protein on monocyte-derived and tissue-resident macrophages, such as microglia of the CNS, it is worth considering the possible implications, if any, of altering wtHTT expression levels when undertaking HTT-lowering as a therapeutic intervention in the disease. Whilst HTT-lowering strategies designed to target mHTT alone are in development, substantial challenges remain^69^. Therefore, non-allele-specific therapies by which expression of both mutant and wild-type forms of the HTT protein are lowered are also being tested, the rationale being that the benefits of lowering mHTT in the context of a fatal, debilitating neurological disease will likely outweigh any negative effects of transiently lowering expression of the wild-type protein. Indeed, significant pre-clinical and clinical work establishing that HTT-lowering is safe and well tolerated has allowed a therapy utilising such a strategy, Tominersen (previously known as IONIS-HTTRx), to be tested in a current phase 3 clinical trial.

If delivery of a drug is restricted to the CNS, any impact on immune system cells in the periphery will be minimal. However, CNS-targeted HTT-lowering could affect microglia in ways both plausibly beneficial in terms of dampening the hyper-reactive inflammatory phenotype of the cells when they express the disease mutation^70^, or less so if loss of the protective effects of wtHTT in microglia means they are less able to deal with the stresses associated with the disease, other insults and/or aging. Any such negative effect, however, might well be outweighed by the overall diminishing of an environment stressful to microglia by the reduced presence of mHTT and its toxic effects. Moreover, the effects of lowering wtHTT in the presence of its mutant counterpart may well differ to those in its absence. Nevertheless, where future HTT-lowering therapeutics are administered systemically such that effects might be seen beyond the degenerating CNS, it would be worthwhile to monitor immune system outcomes in patients to ensure sufficient preservation of innate immune system function in the HTT-lowered condition. More broadly, that wtHTT might well have actions in cells beyond the CNS, as shown here for the first time in normal immune cell health and function, remains a relatively unexplored field of study.

## Methods

### Primary human macrophage cell culture

All experiments with human samples were performed in accordance with the Declaration of Helsinki and approved by the University College London (UCL)/UCL Hospitals Joint Research Ethics Committee (LREC 03/N008). Blood samples were taken from subjects recruited at the UCL Queen Square Institute of Neurology, in accordance with the study’s ethics approval; all subjects provided informed written consent prior to sample donation. Subjects with potential inflammatory or infective conditions were excluded from the study, as were subjects on immunomodulatory medications. Primary human macrophage cell cultures were derived as per previously published methods^31,46,48,49^. Briefly, whole blood samples were collected using BD Vacutainer Cell Preparation Tubes containing polyester gel and a density gradient liquid that allows for isolation of PBMCs through a single centrifugation step at 1000×*g* for 30 min, with the brake turned to “slow” for deceleration. The PBMC layer was then aspirated from the upper interface into a fresh sample tube using a sterile plastic Pasteur pipette. The sample was topped up to 30 ml with ice-cold sterile PBS and centrifuged at 350×*g* for 15 min, and the supernatant discarded. Monocytes were selected by resuspension of the PBMC pellet in 280 µl MACS buffer (PBS, 1% bovine serum albumin (BSA), 2 mM EDTA) and 60 µl anti-CD14 microbeads (Miltenyi Biotec), and incubation at 4 °C for 15 min. The cells-beads mix was then centrifuged at 350×*g* for 5 min and then resuspended in 1 ml MACS buffer. Magnetic cell sorting was carried out by placing pre-washed MACS columns (Miltenyi Biotec) in a magnetic field and passing the cell suspension through. The columns were then washed with a total of 4 ml MACS buffer. Labelled cells were collected by removing the columns from the magnetic field and plunging 2.5 ml MACS buffer through the column twice using a plunger. The sample was topped up to 10 ml with MACS buffer and the isolated monocytes were counted for seeding in culture dishes. Culture of primary human monocytes was carried out in a Containment Level 2 laboratory using strict aseptic technique. They were cultured in R10 media (RPMI 1640 supplemented with 10% FBS, 2 mM L-glutamine, 50 units/ml penicillin and 50 µg/ml streptomycin; Gibco, Thermo Fisher Scientific), using a humidified incubator set to 37 °C with 5% CO_2_. Monocyte-derived macrophages were obtained by adding 20 ng/ml GM-CSF (Cell Guidance Systems) to the monocyte culture media for six days to induce differentiation. Cells were stimulated by treatment with 2 µg/ml LPS (*E. coli* 055:B5; Sigma) and 10 ng/ml IFN-γ (R&D Systems), and stress was induced by treatment with 20 ng/ml bafilomycin A1 (Sigma) for 9 h.

### HTT lowering

β-1,3-D-glucan-encapsulated siRNA particles (GeRPs) were synthesised according to a previously published methods^71^ and loaded with previously validated anti-*HTT* small interfering RNA (Supplementary Table S1)^72^. Macrophages were transfected with GeRP particles on day three of their differentiation protocol, as described previously^46^. GeRPs containing the appropriate siRNA (either targeting *HTT*, or a scrambled siRNA sequence as a control) were added to the cells at a 10:1 particle to cell ratio, then incubated for 24 h before removal by means of a complete media change.

### Quantitative PCR

RNA was isolated using Qiagen’s RNeasy Mini Kit Plus kit, following the manufacturer’s instructions. cDNA conversion was undertaken using Invitrogen Superscript III reverse transcriptase (Thermo Fisher Scientific), also as per the manufacturer’s instructions; qPCR was conducted using Applied Biosystems’ SYBR-Green PCR Master Mix and gene-specific oligonucleotide primers (Supplementary Table S2), including those for endogenous reference genes (*ACTB* and *GAPDH*). Three parallel reactions as technical replicates were measured per single data-point. The ΔΔCt method was applied to report the geometric mean of relative fold-changes of data normalised to that of each reference gene.

### Cytokine multi-plex ELISA

Cytokine profiling was carried out using MesoScale Discovery V-PLEX Proinflammatory Cytokine Panel 1 (Human) ELISA kits, following the manufacturer’s instructions. Supernatants from LPS-stimulated and unstimulated cultures were diluted 1:300 and 1:5, respectively. Three parallel wells as technical replicates were measured per single data-point. Data were normalised to total cell protein content as measured by Pierce BCA assay (Thermo Fisher Scientific), having been washed once with sterile D-PBS and lysed in 60 µl RIPA buffer supplemented with Roche cOmplete Mini EDTA-free protease inhibitor cocktail.

### Imaging flow cytometry

Cells were scraped from their culture dishes in ice cold D-PBS and pelleted by centrifugation at 350×*g* for 5 min. Pelleted cells were fixed and permeabilised using eBioscience intracellular Fix/Perm solutions (Thermo Fisher Scientific). The cells were then incubated in permeabilisation buffer with anti-NFκB p65 XP antibody (Cell Signaling Technology) at 1:200 and 4 °C with shaking for 60 min. Following this, cells were washed twice in FACS buffer (D-PBS with 1% BSA, 0.02% sodium azide) by centrifugation at 350×g for 5 min followed by removal of the supernatant and resuspension in fresh FACS buffer. Cells were then incubated in FACS buffer with eBioscience F(ab')2-Donkey anti-rabbit IgG (H + L) PE-conjugated secondary antibody at 1:100 and 4 °C with shaking for 30 min. After this, cells were washed twice in FACS buffer, then incubated in 1 µg/ml Hoechstt 33342 for 5 min and then washed once before being resuspended in approximately 50 µl FACS buffer for analysis. Single-stained samples were also generated to assess the need for compensation between fluorescent channels. Samples were run on an Amnis ImageStreamX analyser and analysed using IDEAS software version 6.2. Gating was done to isolate in-focus, single cells, that were double-stained for p65 and Hoechstt, and the nuclear localisation wizard in the software allowed gating for translocation events where the p65 stain significantly co-localised with Hoechstt.

### Phagocytosis assay

Monocytes were seeded at 100,000 cells/well of a Cell Carrier-96 plate (Perkin Elmer) and differentiated to macrophages. Following maturation, media were replaced with a serum-free equivalent and incubated overnight to stimulate phagocytosis. Cultures were then washed three times with D-PBS and then incubated with HBSS (with Ca^2+^ and Mg^2+^), 20 mM HEPES for 1 h. Control wells were prepared to allow determination of background fluorescence in a no-cell context. Supernatants were removed and replaced with 100 µl/well resuspended (by sonication for ten 30 s on, 30 off bursts) pHrodo zymosan green or *E. coli* red fluorescent BioParticles (Thermo Fisher Scientific) and the cultures were incubated at 37 °C for 3 h. Wells were then scanned using a microplate reader whereby the fluorescence excitation and emission maxima were 509/533 nm and 560/585 nm for the zymosan green and *E. coli* red particles, respectively. Six parallel wells as technical replicates were measured per single data-point. Phagocytic responses were quantified as a percentage effect calculated as a proportion of the net positive control phagocytosis.

### Viability assay

Cellular LDH (lactate dehydrogenase) levels were analysed using the CytoTox-96 non-radioactive cytotoxicity assay kit (Promega), following the manufacturer’s instructions Monocytes were seeded at 100,000 cells/well of a 96-well plate and differentiated to macrophages. Negative control wells with media only were also prepared to obtain a measurement for background chemiluminescence and maximum LDH release controls were prepared to allow interpretation of the level of LDH as a percentage of maximum LDH for each specific well. Fifty microlitre µl aliquots from all test and control wells were transferred to a fresh 96-well flat clear bottom plate for absorbance (490 nm) measurements on a microplate reader. Three parallel wells as technical replicates were measured per single data-point. LDH levels were quantified as a percentage effect calculated as a proportion of the maximum LDH release measurement for each well.

### Statistical analyses

Data were analysed as the mean of cultures of cells isolated from at least three individual human subjects; the precise numbers of samples used for each experiment are indicated in the figure legends. The overall effect of any given treatment was assessed using paired two-tailed Student’s *t*-tests. A 95% confidence interval (*p* < 0.05) was considered a statistically significant observation. All statistical analyses were conducted using GraphPad Prism 6.07.

## Supplementary information

Supplementary Information.

## Data Availability

All data generated or analysed during this study are included in the published article (and its Supplementary Information files). Full details of the raw data generated and/or analysed and the protocols used during the current study are available from the corresponding authors on reasonable request.
