# Supplementary material for: Wild-type huntingtin regulates human macrophage function
Source: Sci Rep. 2020 Oct 14;10:17269. doi: 10.1038/s41598-020-74042-8 (PMC7560844; doi:10.1038/s41598-020-74042-8)
Supplement: Supplementary file 1 — Supplementary Information. [file 41598_2020_74042_MOESM1_ESM.docx]

**Wild-type huntingtin regulates human macrophage function**

## Authors

Grace C O’Regan, Sahar H Farag, Gary R Ostroff, Sarah J Tabrizi, Ralph Andre

## Supplementary Information

## Supplementary Figure S1.


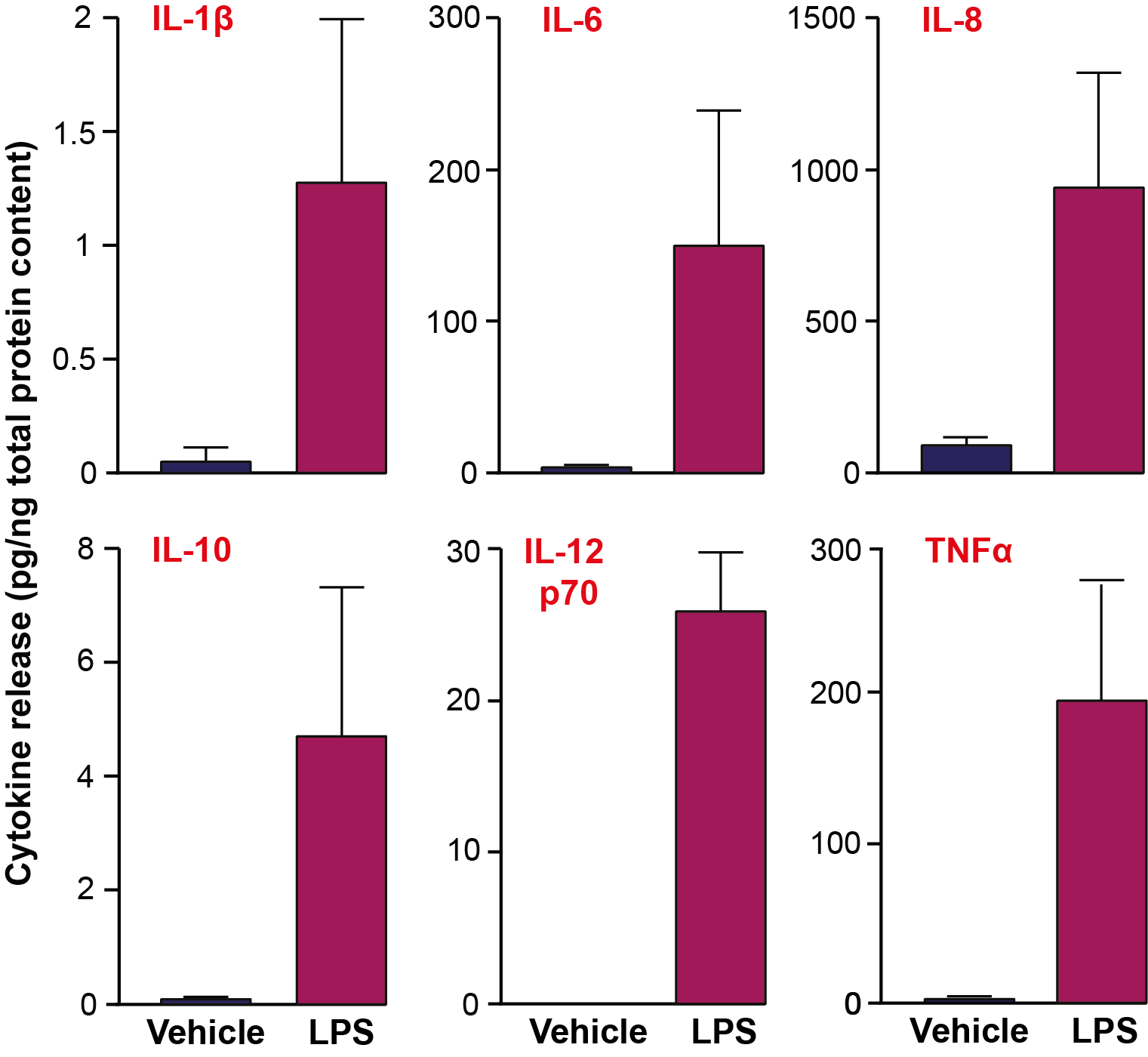


## Supplementary Figure S1. LPS induces cytokine release by human macrophages

## Primary macrophages were derived from blood monocytes harvested from a cohort of healthy human subjects and cultured for treatment with either anti-*HTT* or scrambled siRNA-containing GeRPs. To confirm the stimulating effect of LPS on these cells, supernatants were harvested from cultures following stimulation with 2 µg/ml LPS and 10 ng/ml IFNγ for 24 hours, and analysed by multi-plex ELISA for levels of cytokines relative to total culture protein content, including IL-1β, IL-6, IL-8, IL-10, IL-12 p70 and TNFα.

## Supplementary Table S1.

Sequences of siRNAs used for *HTT* lowering.

| **Target** | | **Sequence** |
| --- | --- | --- |
| anti-*HTT* siRNA | Guide strand | ^5’-^pUUCAUCAGCUUUUCCAGGGUC^-3’^ |
|  | Passenger strand | ^5’-^ CCCUGGAAAAGCUGAUGACGG^-3’^ |
| scrambled siRNA | Guide strand | ^5’-^pUUUCGAAGUACUCAGCGUGAG^-3’^ |
|  | Passenger strand | ^5’-^CACGCUGAGUACUUCGAACUU^-3’^ |

## Supplementary Table S2.

Sequences of oligonucleotide primers used for qPCR.

| **Target gene** | **Forward primer sequence** | **Reverse primer sequence** |
| --- | --- | --- |
| *HTT* | AGTGATTGTTGCTATGGAGCGG | GCTGCTGGTTGGACAGAAACTC |
| *GAPDH* | AACAGCGACACCCACTCCT | CATACCAGGAAATGAGCTTGACAA |
| *ACTB* | AAGGCCAACCGTGAAAAGAT | GTGGTACGACCAGAGGCATAC |
| *IL1B* | CTCAAGTGTCTGAAGCAGCC | GCACTTCATCTGTTTAGGGCC |
| *IL6* | TACCCCCAGGAGAAGATTCC | AGTGCCTCTTTGCTGCTTTC |
| *IL8* | GAGACAGCAGAGCACACAAG | TGCACCTTCACACAGAGCT |
| *IL10* | ATCAAGGCGCATGTGAACTC | GATGCCTTTCTCTTGGAGCTTA |
| *IL12A* | CCTTCACCACTCCCAAAACC | ATTCTAGAGTTTGTCTGGCCTTCT |
| *IL12B* | TTCATCAGGGACATCATCAAACC | ACTCCAGGTGTCAGGGTACT |
| *TNFA* | CCTCAGCCTCTTCTCCTTCC | AGATGATCTGACTGCCTGGG |
